# A set of novel CRISPR-based integrative vectors for *Saccharomyces cerevisiae*

**DOI:** 10.12688/wellcomeopenres.14642.2

**Published:** 2018-07-26

**Authors:** Peter W Daniels, Anuradha Mukherjee, Alastair SH Goldman, Bin Hu

**Affiliations:** 1 Department of Molecular Biology and Biotechnology, The University of Sheffield, Sheffield, UK; 2 Faculty of Life Sciences, University of Bradford, Bradford, UK; 3 Sheffield Institute for Nucleic Acids, The University of Sheffield, Sheffield, UK

**Keywords:** Cas9, yeast, integrative vector

## Abstract

Integrating a desired DNA sequence into the yeast genomes is a widely-used genetic manipulation in the budding yeast *Saccharomyces cerevisiae*. The conventional integration method is to use an integrative plasmid such as pRS or YIplac series as the target DNA carrier. The nature of this method risks multiple integrations of the target DNA and the potential loss of integrated DNA during cell proliferation. In this study, we developed a novel yeast integration strategy based on the widely used CRISPR-Cas9 system and created a set of plasmids for this purpose. In this system, a plasmid bearing Cas9 and gRNA expression cassettes will induce a double-strand break (DSB) inside a biosynthesis gene such as Met15 or Lys2. Repair of the DSB will be mediated by another plasmid bearing upstream and downstream sequences of the DSB and an integration sequence in between. As a result of this repair the sequence is integrated into genome by replacing the biosynthesis gene, the disruption of which leads to a new auxotrophic genotype. The newly-generated auxotroph can serve as a traceable marker for the integration. In this study, we demonstrated that a DNA fragment up to 6.3 kb can be efficiently integrated into the Met15 or Lys2 locus using this system. This novel integration strategy can be applied to various yeasts, including natural yeast isolated from wild environments or different yeast species such as *Candida albicans*.

## Introduction

The budding yeast *Saccharomyces cerevisiae* has been widely used as a model organism for basic research in all aspects of eukaryotic biology. With at least 31% of *S. cerevisiae*’s more than 6000 genes having homologues in the human genome ^1^, the insight gained from yeast-focused investigation greatly benefits our understanding of the molecular basis of human pathogenesis, such as cancer and Alzheimer’s disease.

There are many versatile and powerful tools available for the yeast genome manipulation, which contribute to the popularity of yeast as a model organism. For example, a DNA fragment can be integrated by homologous recombination (HR) into the yeast genome, allowing the expression of an exogenous gene or modified yeast gene. A traditional integration method of choice, namely the YIplac-series ^2^ or pRS-series ^3^ integrative vectors, has been successfully used to this end for several decades. An integrative plasmid typically uses an *E. coli* cloning vector as the backbone and contains a yeast nutritional marker, normally a yeast biosynthesis gene. The most commonly used nutritional markers are the *URA3*, *LEU2*, *TRP1*, and *HIS3* genes, which are required for synthesis of pyrimidine, L-leucine, L-tryptophan, and L-histidine respectively. To integrate these vectors into yeast genome, these circular plasmids are first linearized by restriction digestion occurring inside the nutritional marker genes. After transformation, the linearized vectors can be integrated into the nutritional marker gene locus in the yeast genome through HR. It is important that the host yeast cells should harbor the corresponding auxotrophic mutation or deletion (also called auxotrophic marker) for the selection of the successful transformants. The consequence of this integration is the target DNA sequence being flanked by a mutated allele and a wild-type allele of the nutritional marker. Therefore, the yeast cells acquiring the integrative vector become prototrophic for the corresponding nutritional marker ^4^.

Several drawbacks of these systems have been known to limit its application. First, the corresponding auxotrophic marker is required for the selection of transformants. For example, the *LEU2* integrative plasmid only can be used in cells with a *leu2* auxotrophic marker, which is unavailable in some yeast laboratory strains. More seriously, a yeast strain isolated from a wild environment would lack all of the available auxotrophic markers, which makes the application of this system impossible in these cells. Thirdly, the short supply of existing nutritional markers limits the application of this commonly used system in some laboratory strains. Moreover, the integrated DNA sequence flanked by the wild-type and mutant genes can be looped out from yeast genome by random recombination between these two homologous sequences ^4^. This event can result in leaving behind either the wild-type or mutant gene on the chromosome depending on the site where the recombination occurs. In this case, a prototroph genotype of offspring arising from the original transformant would not guarantee the maintenance of the integrated DNA sequence in the genome. Finally, the manner of this integration can lead to tandem repeats of the sequence, which can cause undesirable overexpression of the target gene. To circumnavigate these problems, new strategies have been developed. For example, a DNA fragment containing the integration DNA sequence plus an antibiotic marker can be inserted into the yeast genome by replacing the *leu2* or homothallic switching endonuclease (HO) sequence ^5, 6^. Although this method avoids multi-integration and instability of integration, it requires an extra antibiotic marker. Furthermore, including the extra sequence for the antibiotic marker will restrict the size of the integration sequence since the integration efficiency of a longer DNA sequence is reduced.

The CRISPR-Cas9 system has been extensively used to modify the genome in eukaryotes including yeast ^7, 8^. It is composed of a Cas9 nuclease that generates a DNA double strand break (DSB), and a guide RNA (gRNA) that precisely recruits the Cas9 nuclease to a target DNA sequence. By harnessing this system, a strategy for a marker-free integration involving a template DNA sequence can be used for the repair of the DSB. As a result of this repair, the desired DNA sequence is integrated into the DSB site ^9^. This method avoids all of the mentioned limitations for conventional integration approaches. However, the integration is difficult to trace in later genetic manipulations due to the lack of a genetic marker. We present here a set of CRISPR-Cas9 plasmids and corresponding repair plasmids that make integration more practical, flexible and traceable. The CRISPR-Cas9 plasmid is used to introduce a single DSB in a gene encoding an essential biosynthesis enzyme. The repair plasmid provides a linear target DNA sequence flanked by sequences homologous to each side of the DSB, which is used as a template sequence for homologous repair (HR) of the DSB. As a consequence, the biosynthesis gene is replaced with the integrating DNA sequence. This leads to a new auxotrophic genotype, which can be used as a genetic marker for the integration. In this study, we constructed two sets of CRISPR-based yeast integration systems targeting Lys2, an α-aminoadipate reductase for biosynthesis of lysine, and Met15, an O-acetyl homoserine-O-acetyl serine sulfhydrylase for biosynthesis of methionine. We also demonstrated the efficient integration achieved by these systems.

## Methods

### Yeast and *Escherichia coli* culture

All yeast strains used in this study were derived from W303 (Mat a, *ade2-1*, *trp1-1*, *can1-100*, *leu2-3,112*, *his3-11,15*, *ura3-52*). Yeast cells were grown in rich medium (yeast extract peptone; YEP) supplemented with 2% glucose (yeast extract peptone dextrose; YPD) or synthetic complete (SC) medium at 30°C. Cultures were agitated at 200rpm (Kühner Shaker). YPD-Nat plates contained YEP medium with 2% glucose and 100 µg/ml clonNAT (Catalog No N5375-74, Stratech Scientific, UK). *E. coli* strain XL1-Blue (Catalog No 200249, Agilent Technologies) was used for plasmid manipulation. The *E. coli* standard media and transformation were described as previously ^10^.

### Plasmid construction

The plasmids expressing gRNAs targeting Lys2 and Met15 were derived from the plasmid pBH257 (a gift from Prof. Kim Nasmyth’s lab). The pBH257 is a pRS425-based plasmid bearing a Cas9 endonuclease expression cassette and a *SNR52* promoter-controlled gRNA-encoding sequence. This plasmid also contains two *Sap*I restriction endonuclease sites downstream of the *SNR52* promoter for insertion of a 20bp targeting DNA sequence. To construct the CRISPR plasmids targeting Lys2 or Met15, a pair of oligonucleotides (5µM each in 50mM NaCl solution) with complementary targeting sequences were annealed by incubation in a PCR machine (ProFlex™ PCR System, Life technology) at the following temperatures: 95°C for 2min, 72°C for 2min, 50°C for 2min, and 25°C for 2min. The annealed DNA was ligated with the *Sap*I-digested pBH257 plasmid (Catalog No 6022, Takara DNA Ligation Kit, Version 2.1, Clontech) and transformed into *E. Coli* as described previously ^10^.

To construct the repair plasmids for the repair of the DSB occurring inside Lys2 or Met15, two 1 kb DNA fragments containing the upstream and downstream sequences of Lys2 or Met15 were amplified by PCR and cloned into pBlueScript SK- plasmid. To facilitate the cloning of a DNA sequence into the repair plasmid, a multi-cloning site was introduced between these two homologous sequences. A pair of *Not*I and *Pae*I restriction endonuclease sites were also inserted at the 5’ end of the upstream homologous sequence and at the 3’ end of the downstream homologous sequence, which can be used to release the repair fragment from the plasmids.

### Yeast transformation

Yeast transformation was performed as previously described ^11^. Briefly, yeast cells, exponentially grown, were pelleted and suspended to a final cell density of 4 × 10 ^9^ cells/ml with 100mM lithium acetate. Aliquots (50μl) were pre-incubated at 30°C for 30 minutes and mixed with 240μl of 50% (w/v) Polyethylene Glycol (PEG), 36μl of 1.0M lithium acetate, 25μl of single stranded DNA, and 50μl DNA mixture. The DNA mixture contains 2.5µg of Cas9-targeting plasmid and 5µg of *Not*I or *Pae*I-digested repair plasmid. The cell mixture was incubated for 30 min at 30°C and heat shocked at 42°C for 20 min. Cells were then harvested by centrifugation in a microfuge (3,000×g; 3 min) and gently resuspended in 7ml of YPD. After incubation for 90 minutes at 30°C while gently shaking (100rpm), the cells were spread onto appropriate agar plates and incubated at 30°C for 2–3 days.

### Quick DNA extraction and diagnostic PCR

The genome DNA used for diagnostic PCR was extracted by a LiOAc/SDS method ^12^. The yeast cells grown overnight on YPD plates were collected using 1µl inoculation loops and suspended in 100μl of lysis solution (200 mM LiOAc and 1% SDS). The cells were lysed at 70°C for 10 min. The DNA was precipitated by the addition of 300μl of 100% ethanol and collected by centrifugation (14,500 g× 5 minutes). Supernatant was removed and the pellet was washed with 500 μl of 70% ethanol. The DNA was air-dried in an extraction hood for 15 minutes and dissolved in 100μl of MiniQ water. The debris was removed in a micro-centrifuge (14,500 g× 5 minutes) and 1μl of supernatant was used for a PCR reaction with a final volume of 20 μl. The PCR reaction was performed under recommended conditions (Catalog No PB10.11, PCRBIO HiFi Polymerase, PCR Biosystem). The 20 μl reaction mixture consists of l × PCR buffer containing 3mM of MgCl _2_ and 1 mM of dNTP, 400 nM of each primer and 1 unit of Taq DNA polymerase. Thermo cycle condition was: Initial denaturation at 95°C for 2 min followed by 30 cycles of denaturation at 95°C for 15 seconds, annealing at 55°C for 15 seconds and extension at 72°C for 30 seconds. The sequences for the primers used in this study were listed in Table 1. Primers were sourced from Integrated DNA Technologies.

**Table 1.** Primers used in this study.

Primer used in this study	Sequence
Lys2Cas9F	ATCCTTGAGTAGGGACATACAAT
Lys2Cas9R	AACATTGTATGTCCCTACTCAAG
Met15cas9F	ATCGATACTGTTCAACTACACGC
Met15cas9R	AACGCGTGTAGTTGAACAGTATC
Met15OutF	ATTTGCGTCATCTTCTAACACCG
Met15OutR	TATTATGGCCTCTAGCAGCAACG
Lys2outF	AGACCCGCTGGGAGAAGTTCAAG
Lys2outR	ATGCTCAACCTTAAGCTGCTGCG
NatF	GACCGTCGAGGACATCGAGGTCG
NatR	TCGTCGGGGAACACCTTGGTCAG

## Results and discussion

### The strategy for the Cas9-mediated integration

**Figure 1.**
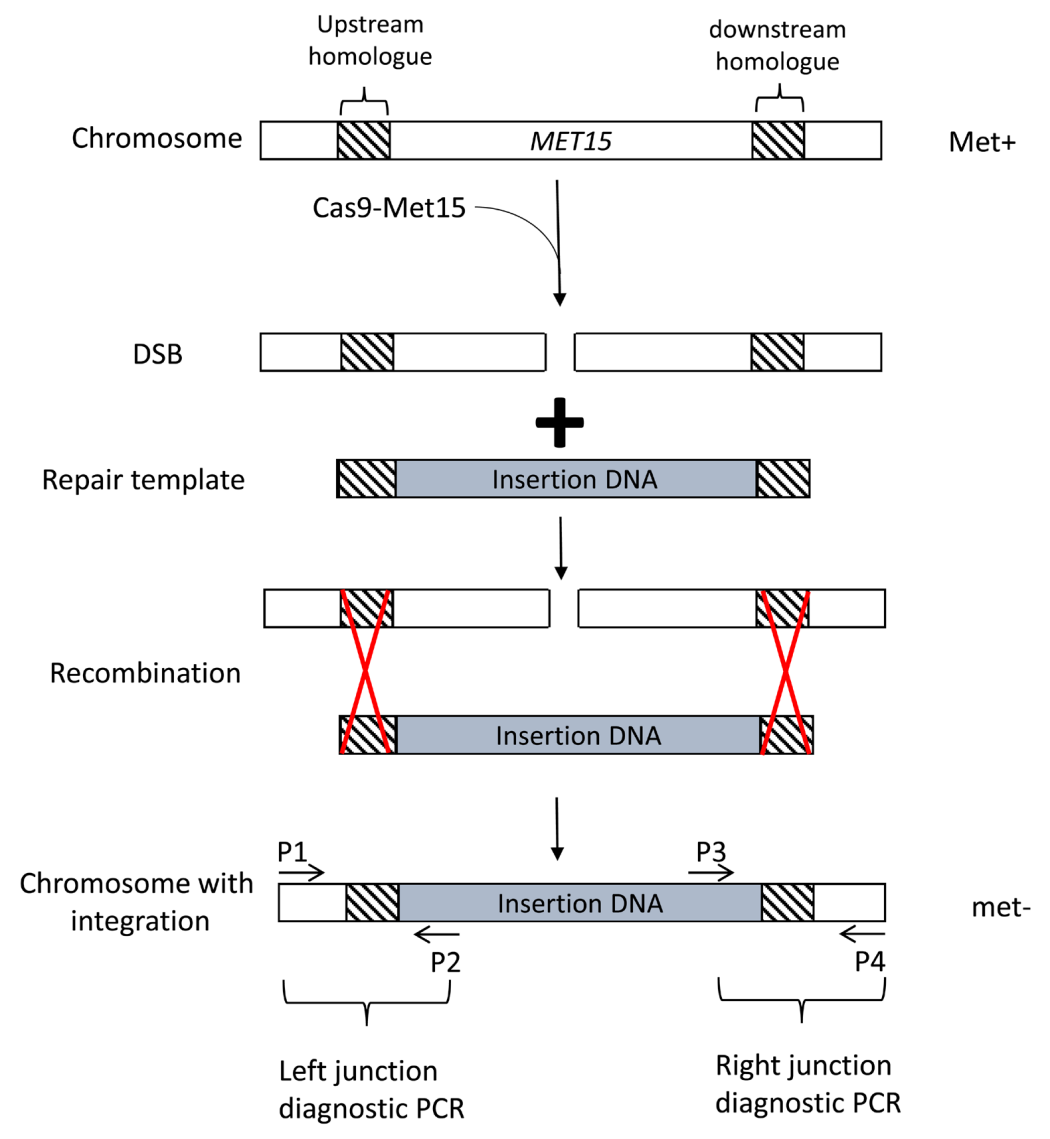
Flow scheme of the Cas9-mediated integration procedure.

**Figure 2.**
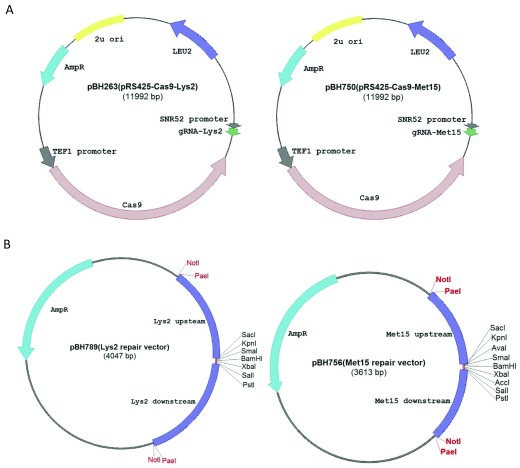
Physical maps of the Cas9/gRNA plasmids ( **A**) and the repair plasmids ( **B**). The positions of the yeast and *Escherichia coli* selection markers and multi cloning sites are indicated.

In this study, we developed a novel integration system in *Saccharomyces cerevisiae*, which is based on the CRISPR-Cas9 system. The principle of this system is that the desired DNA sequence is integrated into the yeast genome as a consequence of homologous repair of a DSB generated at a specific site by the CRISPR-Cas9 system. To facilitate marking of the integration, the DSB site is designed to occur at a gene encoding a key enzyme in a biosynthesis pathway ( Figure 1). In this study we chose Lys2 and Met15 as integration sites, which are essential enzymes for the synthesis of lysine and methionine. To do this, we constructed two pRS425-based episomal plasmids, pBH263 and pBH750, each bearing expression cassettes for Cas9 and Lys2/Met15-targeting gRNA ( Figure 2A). When these plasmids were introduced into yeast cells, a DSB was produced inside the Lys2 or Met15 gene by Cas9. To repair this DSB, a template DNA consisting of the upstream and downstream sequences of Lys2/Met15 and the integration sequence in between, was co-transformed with the Cas9 plasmid. It has been reported that the Cas9-produced DSB can be repaired with a template DNA bearing short homologous sequences of 50 bp, which can be introduced by PCR with primers containing these sequences. In this study, we used longer homologous sequences (around 400–500 bp) to improve the integration efficiency. For this purpose, we constructed two repair plasmids, pBH756 and pBH789, which contain two long homologous sequences for HR repair and multi cloning sites for the insertion of the integration sequence ( Figure 2B). To release the repair template DNA from these vectors, two *Not*I/ *Pae*I digestion sites were introduced at the both ends of the repair cassette. This strategy avoids PCR amplification of the repair template DNA, which could introduce mutations and also limits the length of the integration fragment. As a result of the DSB repair using the repair fragment as the template, the target DNA sequence is integrated into the yeast genome by replacing *MET15* or *LYS2* gene. This integration can be PCR-verified using two pairs of primers as shown in Figure 1. The cells with successful integration become auxotrophic for lysine or methionine. This irreversible auxotrophic genotype completely genetically links the integrated DNA, so it can be used as a reliable marker for the integration.

### The integration efficiency of the Cas9-mediated integration

To test the efficiency of Cas9-mediated integration, we used *NatMX*, a dominant nourseothricin-resistance marker, as the target DNA sequence. This sequence was cloned into the repair plasmids pBH756 and pBH786. The repair template fragment bearing the homologous sequences was released by *NotI*-digestion and co-transformed into yeast cells (K699) with the Cas9/gRNA *LEU2* plasmid (pBH263 or pBH750). The cells containing the vectors were selected by growing on SC plates omitting leucine. Using the standard yeast transformation protocol described in the methods section, around 400–500 *LEU*-positive colonies were obtained in one transformation reaction. To achieve a high co-transformation efficacy of the template DNA with the Cas9 plasmid, a molecule ratio of 10:1 of *NatMX* DNA to Cas9 plasmid was used in the transformation reaction. In this context, we expected that the yeast cells with successful integrations would become auxotrophic for methionine or lysine (Lys- or Met-) and gain a nourseothricin-resistance (NAT+) genotype. To test this, we picked 60 transformants randomly from the *MET15*-targeted or *LYS2*-targeted integrations and determined their genotypes. This was achieved by examining their growth on SC plates omitting methionine/lysine or YPD plates containing nourseothricin. As shown in Table 2 and Table 3, more than 90% of transformants displayed NAT+ phenotype, suggesting a high co-transformation efficiency of *NatMX*. Surprisingly, around 20–40% of these transformants were still methionine or lysine prototrophic (Met+ or Lys+). In these cells, the Cas9 system might have failed to generate a DSB. Another explanation is that the DSB repair might have produced a mutated allele of Met15 or Lys2 with partial enzyme activity still being sufficient for the biosynthesis of methionine or lysine. The question was then how the NatMX DNA was maintained in the cells since this linear DNA fragment lacks a replication origin (ARS). One possibility that this NatMX fragment acquired an ARS from genome and self-circularised. In this case, the NAT+ genotype will be genetically unstable. To test this, we crossed ten independent Lys+ NAT+ transformants with a wild-type MATα strain (K700). We found that all the spores derived from these crosses lost the NAT+ phenotype (unpublished study, Daniels PW and Hu B). This suggested that the NatMX DNA in these cells was not integrated into the yeast genome and therefore could be lost during mitosis or meiosis.

**Table 2.** Genotypes of transformants obtained from the co-transformation of Csa9-Met15 and NatMX cassette.

Genotype	Number of transformants	%
*Nat resistant,* *met-*	48	80.0%
*Nat resistant,* *Met+*	10	16.7%
*Nat sensitive,* *met-*	0	0%
*Nat sensitive,* *Met+*	1	1.7%
**Total**	**60**	

**Table 3.** Genotypes of transformants obtained from the co-transformation of Csa9-Lys2 and NatMX cassette.

Genotype	Number of transformants	%
*Nat resistant,* *lys-*	34	56.7%
*Nat resistant,* *Lys+*	23	38.3%
*Nat sensitive,* *lys-*	0	0%
*Nat sensitive,* *Lys+*	6	10.0%
**Total**	**60**	

**Figure 3.**
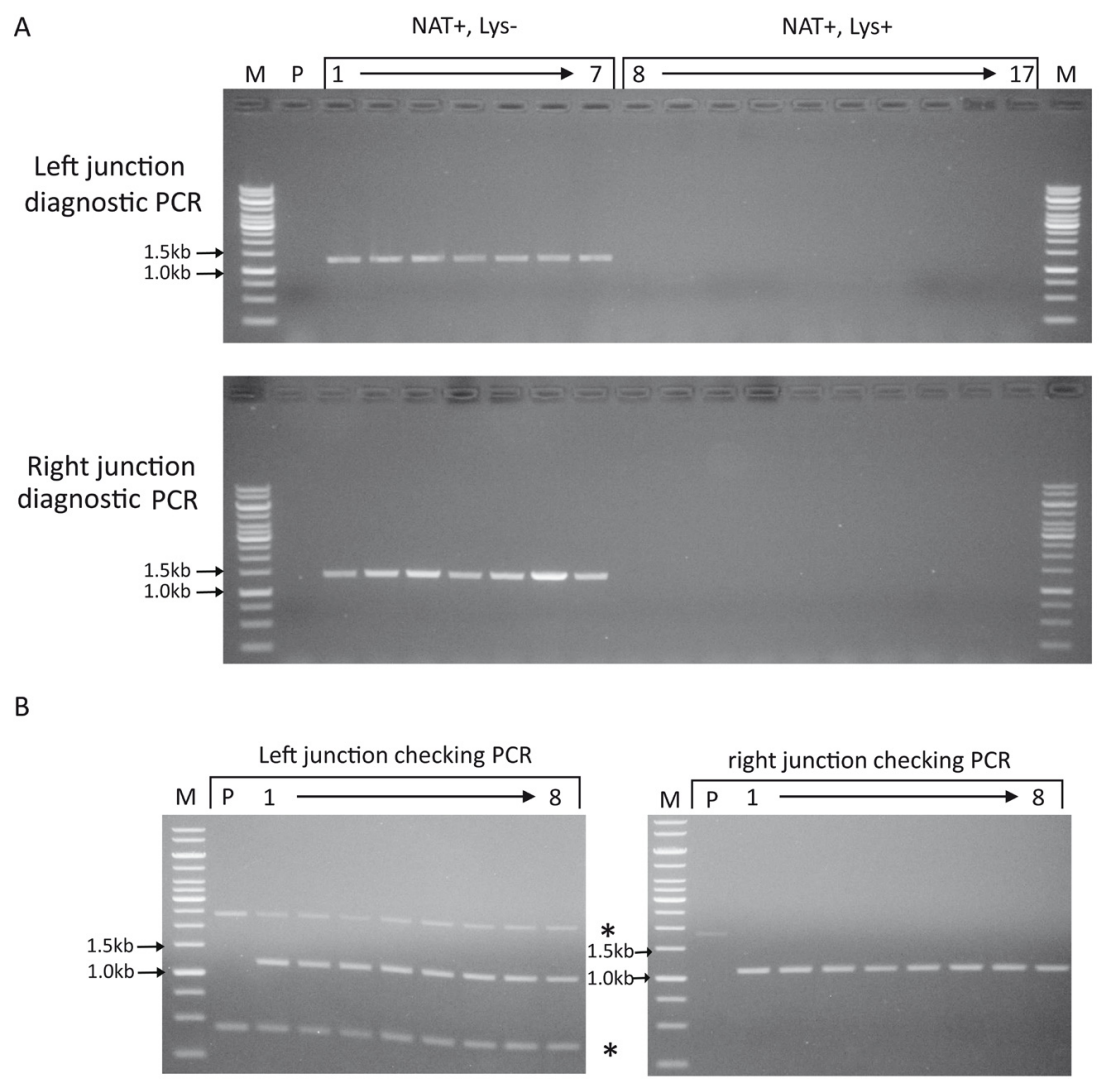
Diagnostic PCR confirm the integration of a NatMX cassette. ( **A**) Yeast cells (K699) were co-transformed with the Cas9-Lys2 plamid (pBH263) and the Not1-disgested NAT cassette (pBH762), designed to be integrated into the Lys2 locus. The transformants were selected by plating on SD-LEU plates. Genomic DNA was isolated from the parental yeast K699 (P) and the transformants with the indicted genotypes. The PCR reactions were carried out using the primer pairs Lys2outF/NatR for checking left junction (top panel) and NatF/Lys2outR for checking right junction (low panel). The expected sizes of diagnostic PCR are 1.3 kb for left junction and 1.4 kb for right junction. ( **B**) Eight NAT+ Met- transformants derived from the co-transformation of the Cas9-Met15 plasmid (pBH750) and the Not1-disgested NAT cassette (pBH763) were selected for diagnosis PCR. The primer pairs Met15outF/NatR for checking left junction (left panel) and NatF/Met15outR for checking right junction (panel panel) were used in the PCR reaction. The expected sizes of diagnostic PCR are 1.2 kb for left junction and 1.1 kb for right junction. *indicated non-specific amplification.

We also performed tetrad analysis to examine the genetic stability of the NAT+ genotype in the Lys- transformants and the linkage of NAT+ with *lys2*. As expected, the segregation of the NAT+ genotype for every tetrad derived from 10 crosses showed a 2:2 ratio and Lys- co-segregated with the NAT+ genotype in all the cases. This indicated that the NatMX cassette was integrated into the Lys2 locus, which led to its disruption. To further confirm this, we carried out diagnostic PCR to examine the right and left junctions resulting from the integration using the primer pairs Lys2OutF/NatR and NatF/Lys2outR ( Figure 1). The expected DNA products were successfully amplified from all eight Lys- NAT+ transformants, revealing correct integrations in these cells ( Figure 3A). These amplifications failed in the Lys+ NAT+ cells, which was consistent with the notion that the NatMX cassette failed to be integrated into Lys2 locus ( Figure 3A). We also used the same strategy to confirm the expected integration of the NatMX cassette into the Met15 locus in eight NAT+ Met- transformants obtained from the NatMX cassette and pBH750 co-transformation. These results suggested the transformants that gained the auxotrophic genotype were very likely to possess the integrated DNA at the expected site. The acquired auxotroph can be used a reliable marker to trace the integration in later genetic manipulations.

**Figure 4.**
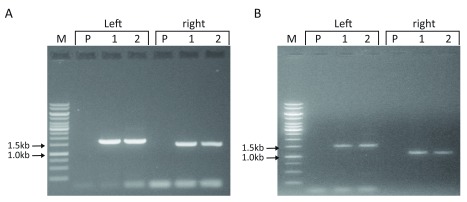
Diagnostic PCR products that confirm the integration of a 6.3kb Smc3 cassette. ( **A**) Yeast cells (K699) were co-transformed with the Cas9-Lys2 plasmid (pBH263) and the Not1-disgested Smc3 cassette (pBH767) and diagnostic PCR were carried out with Lys- LEU+ transformants. The primer pairs Lys2outF/Smc3R and Sm3F/Lys2outR were used for checking left and right junction. The expected sizes of diagnostic PCR are 1.7 kb for left junction and 1.4 kb for right junction. ( **B**) Yeast cells (K699) were co-transformed with the Cas9-Met15 plasmid (pBH750) and Not1-disgested Smc3 cassette (pBH766) and diagnostic PCR were carried out in Lys- LEU+ transformants. The primer pairs Met15outF/Smc3R and Sm3F/Met15outR were used for checking left and right junction. The expected sizes of diagnostic PCR are 1.5 kb for left junction and 1.1 kb for right junction.

In this study, we have demonstrated the application of our Cas9/gRNA system for successful integration of a 1.2 kb NatMX cassette into the Met15 or Lys2 loci. But, on many occasions, a larger fragment is required to be integrated into the yeast genome. To explore this possibility, we made an attempt to integrate a DNA fragment with a size more than 6kb. We first inserted a 6.3kb DNA fragment containing the Smc3 encoding sequence plus their full promoter and terminator into the repair vectors. After co-transformation of the *Not*I-released repair template with the Cas9/gRNA plasmids, around 300–400 transformants were obtained on SC –Leu plates. Out of sixteen chosen colonies, only around 20% of the transformants acquired the expected auxotrophic genotype, suggesting a reduced efficiency for the integration of a larger DNA fragment. This could be due to the larger DNA being prone to degradation in cells. We confirmed that the Lys- or Met- transformants also had successful integrations using diagnostic PCR, which produced DNA fragments with the expected sizes ( Figure 4).

### Removal of the Cas9 plasmid

The remaining Cas9/gRNA plasmid in cells with successful integrations could affect further genetic operation. For example, it could produce undesired DSB during a cross with other cells containing wild type *LYS2* or *MET15*. The existence of these *LEU2* plasmids would prevent the usage of leu2- auxotrophic markers in future genetic manipulation. Here, we developed a simple method to remove the Cas9/gRNA plasmid from the successful transformants. The cells with the integration of the target DNA were grown in YPD liquid media overnight. The diluted culture was then spread onto a YPD plate to allow the formation of 300–500 colonies. These cells were then replica plated onto SC –Leu solid media to determine the colonies losing the Cas9/gRNA plasmid. Our data showed that more than 80% cells lost this vector after an overnight culturing (unpublished study, Daniels PW and Hu B).

## Conclusion

In this study we developed a Cas9-based genome integration system in *Saccharomyces cerevisiae* that overcomes the drawbacks of conventional integration methods. To simplify the procedure and enhance the flexibility of integration to a great extent, we created a set of plasmids including the Cas9/gRNA and the corresponding repair vectors. We have demonstrated the successful integration of target DNA fragments of a size up to 6.3kb. Though only two sites, Lys2 and Met15, were tested in this study, many other sites harboring key biosynthesis enzymes encoding sequences could be used in this system. This would greatly expand available integration sites. Furthermore, since the Cas9 system has been proved to work efficiently in many eukaryotic organisms, we are optimistic that such a strategy could be used in other popularly-studied yeasts, especially those lacking sufficient genetic markers, such as *Candida albicans*.

## Data availability

The data underlying this study is available from OSF. Dataset 1: A set of novel CRISPR-based integrative vectors for Saccharomyces cerevisiae.

DOI http://doi.org/10.17605/OSF.IO/MRT86 ^13^.

This dataset is available under a CC0 1.0 Universal license.
