# Supplementary material for: A set of novel CRISPR-based integrative vectors for Saccharomyces cerevisiae
Source: Wellcome Open Res. 2018 Jul 26;3:72. Originally published 2018 Jun 15. [Version 2] doi: 10.12688/wellcomeopenres.14642.2 (PMC6051238; doi:10.12688/wellcomeopenres.14642.2)
Supplement: Peer review history

# Peer review: Referee response for version 2

**DOI:** 10.21956/wellcomeopenres.16021.r33581

**Published:** 2018-07-27

**Authors:** Barry Panaretou

**Affiliations:** 1 School of Cancer & Pharmaceutical Sciences, King's College London, London, UK

All of my concerns were addressed. The article describes a new way of making precise changes to the yeast genome, and advantages over previously described methods have been emphasized.

I have read this submission. I believe that I have an appropriate level of expertise to confirm that it is of an acceptable scientific standard.

# Peer review: Referee response for version 1

**DOI:** 10.21956/wellcomeopenres.15943.r33364

**Published:** 2018-07-17

**Authors:** Barry Panaretou

**Affiliations:** 1 School of Cancer & Pharmaceutical Sciences, King's College London, London, UK

This is a useful addition to the molecular genetics toolkit for *Saccharomyces cerevisise*.

 Points that should be dealt with:

 Figures 3 and 4  present results of diagnostic PCR reactions.  However, the expected sizes of PCR fragments are not stated in the text (also, the molecular weight markers migrating above and below the relevant PCR products should be labelled with their size).

 The CRISPR/Cas9 system of Mans et al. (2015) ^1^ should be cited. Any differences between that system and the one described in this manuscript, should be emphasized.

 Minor points:

 In the abstract:

 line 1, 'DNA sequence into **the** yeast genome' (add 'the')...same applies in latter half of abstract ' ...the sequence is integrated into ' **the**' genome' (and a similar correction in the 2nd paragraph of the introduction).

 'homologue repair' should be replaced with 'homologous repair' (wherever this appears, e.g. page 3 column 2).

 'homologue sequences' should be replaced with 'homologous sequences', wherever this phrase appears (multiple times throughout the manuscript).

 On diagram at top of page 5, 'homolog' should be replaced by 'homologue'.

 'described' used twice at end of paragraph on page 4.

 At end of page 6, column heading should be 'Number of transformants'.

I have read this submission. I believe that I have an appropriate level of expertise to confirm that it is of an acceptable scientific standard, however I have significant reservations, as outlined above.

## References

[1] MansRvan RossumHMWijsmanMBackxAKuijpersNGvan den BroekMDaran-LapujadePPronkJTvan MarisAJDaranJM: CRISPR/Cas9: a molecular Swiss army knife for simultaneous introduction of multiple genetic modifications in Saccharomyces cerevisiae. *FEMS Yeast Res*.2015;15(2) : 10.1093/femsyr/fov004 10.1093/femsyr/fov004 25743786PMC4399441

# Peer review: Referee response for version 1

**DOI:** 10.21956/wellcomeopenres.15943.r33366

**Published:** 2018-07-03

**Authors:** Stefan H. Millson

**Affiliations:** 1 University of Lincoln, Lincoln, UK

The article is a sound piece of research methodology. The need for new cloning tools in the yeast field has been highlighted in the article, as a lot of currently used yeast vector systems are over 20 years old and should be replaced – this paper goes part way to assisting with solving this problem.

 This paper is timely as the need for next generation yeast cloning systems to catch-up with the array of mammalian systems available is great. I found the paper to have the depth of methodology and a refreshing honesty about the results – I totally recommend for expedient indexing.

 A few very minor suggested alterations;

 The title is not that descriptive - maybe change to a title that might to excite the reader a little more?

 Third paragraph of the introduction change “this system” to “these systems” as more than one system was discussed.

 Same paragraph change “overcome” to “circumnavigated” as these problems were not overcome but in reality avoided.

 Change centrifuge speed in methods from rpm to x g.

 Methods Taq amount would be best to quote amount as units not volume.

I have read this submission. I believe that I have an appropriate level of expertise to confirm that it is of an acceptable scientific standard.

# Peer review: Referee response for version 1

**DOI:** 10.21956/wellcomeopenres.15943.r33365

**Published:** 2018-07-02

**Authors:** Olivier Gadal

**Affiliations:** 1 Laboratory of Eukaryotic Molecular Biology (LBME), Center for Integrative Biology (CBI), National Centre for Scientific Research (CNRS), UPS, University of Toulouse, Toulouse, France

Daniels *et al.* made an innovative yeast integration method based on CRISPR-cas9 system. This method allows integration of a sequence of interest at the MET15 or LYS2 gene. For this purpose the authors developed a set of plasmids. They built plasmids coding for the endonuclease Cas9 and guide RNAs.  Expression of Cas9 and guide RNAs in yeast allow the induction of double strand break inside the gene MET15 or LYS2.  Repair of those double strand breaks can be made by transformation yeast with another plasmid bearing upstream and downstream sequence of the double strand break and the sequence of interest between. Repair of this DSB allows integration of the interest sequence and consequently induces inactivation of the MET15 or LYS2.  Disruption of those genes creates an auxotrophic genotype that can be used for follow the integrated gene. The authors provide a good explanation of the plasmid construction in the materials and method. The clearly demonstrated that it is possible to integrate with their new developed system DNA sequence up to 6.3 kb.  

 Integrative vectors  (YIplac or pRS vectors) have been used for several years. They allow the integration of a sequence of interest into the URA3, LEU2, TRP1 or HIS3. Although those vectors are commonly used in the laboratories their applications are limited. Multiple integration at the autotrophic marker can induced undesirable over expression of the interest gene. Moreover, an auxotrophic marker is required for the selection of transformants.  Method developed by Daniels *et al*. allows overcoming the problem of undesirable over expression as it allows only single integration.  Moreover, Daniels *et al *technology offers the possibility to integrate DNA sequence in yeast isolated from a wild environment, which lack all auxotrophic markers.

 I think that this method will be useful for the yeast community. Therefore, I recommend strongly this work for indexing.

I have read this submission. I believe that I have an appropriate level of expertise to confirm that it is of an acceptable scientific standard.
